# A Novel Method to Efficiently Highlight Nonlinearly Expressed Genes

**DOI:** 10.3389/fgene.2019.01410

**Published:** 2020-01-31

**Authors:** Qifei Wang, Haojian Zhang, Yuqing Liang, Heling Jiang, Siqiao Tan, Feng Luo, Zheming Yuan, Yuan Chen

**Affiliations:** ^1^ Hunan Engineering & Technology Research Center for Agricultural Big Data Analysis & Decision-Making, Hunan Agricultural University, Changsha, China; ^2^ School of Information Science and Technology, Hunan Agricultural University, Changsha, China; ^3^ School of Computing, Clemson University, Clemson, SC, United States

**Keywords:** RNA sequencing, maximal information coefficient, differential expressed gene, gene selection, normalized differential correlation

## Abstract

For precision medicine, there is a need to identify genes that accurately distinguish the physiological state or response to a particular therapy, but this can be challenging. Many methods of analyzing differential expression have been established and applied to this problem, such as *t*-test, edgeR, and DEseq2. A common feature of these methods is their focus on a linear relationship (differential expression) between gene expression and phenotype. However, they may overlook nonlinear relationships due to various factors, such as the degree of disease progression, sex, age, ethnicity, and environmental factors. Maximal information coefficient (MIC) was proposed to capture a wide range of associations of two variables in both linear and nonlinear relationships. However, with MIC it is difficult to highlight genes with nonlinear expression patterns as the genes giving the most strongly supported hits are linearly expressed, especially for noisy data. It is thus important to also efficiently identify nonlinearly expressed genes in order to unravel the molecular basis of disease and to reveal new therapeutic targets. We propose a novel nonlinearity measure called normalized differential correlation (NDC) to efficiently highlight nonlinearly expressed genes in transcriptome datasets. Validation using six real-world cancer datasets revealed that the NDC method could highlight nonlinearly expressed genes that could not be highlighted by *t*-test, MIC, edgeR, and DEseq2, although MIC could capture nonlinear correlations. The classification accuracy indicated that analysis of these genes could adequately distinguish cancer and paracarcinoma tissue samples. Furthermore, the results of biological interpretation of the identified genes suggested that some of them were involved in key functional pathways associated with cancer progression and metastasis. All of this evidence suggests that these nonlinearly expressed genes may play a central role in regulating cancer progression.

## Introduction

Identifying and characterizing biomarkers that accurately reflect a physiological state (normal or diseased) or response to a particular drug or therapy is challenging. High-quality biomarkers are especially important for cancer detection and the development of safe, effective treatments. Such biomarkers are also the ultimate goal of many next-generation sequencing studies (Xiao et al., 2017; American Association for the Advancement of Science, 2018).

**Figure 1:**
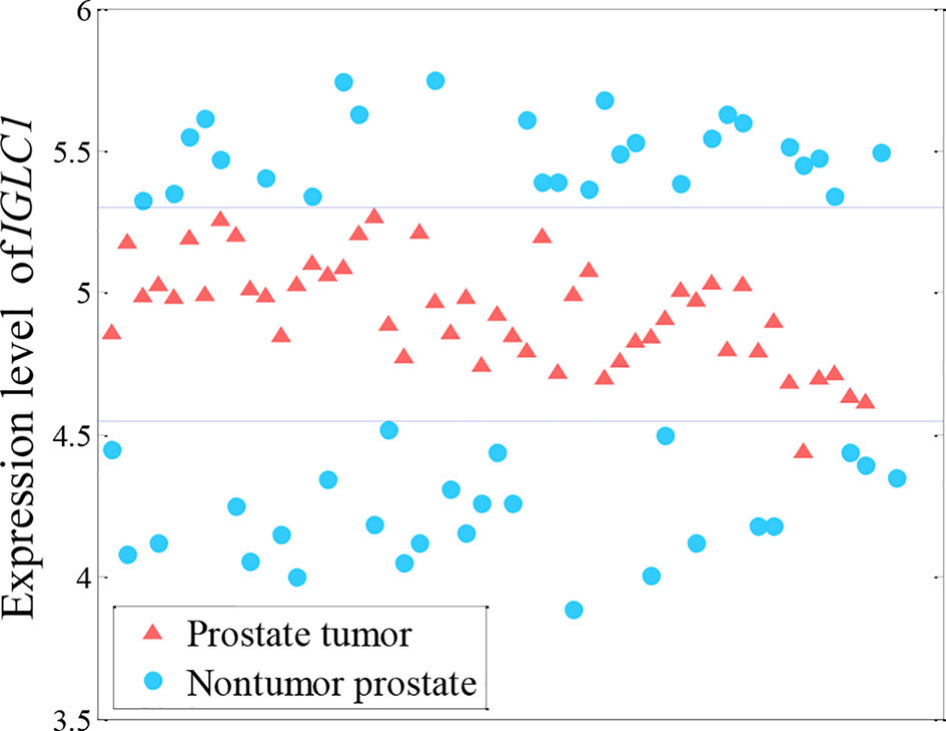
Nonlinearly expressed pattern of the *IGLC1* gene for prostate tumor from microarray data (Singh et al., 2002). Both high and low levels of *IGLC1* gene expression are found in non-tumor samples.

Many methods for analyzing differential expression in RNA sequencing (RNA-seq) data with the aim of finding genes that are differentially expressed across groups of samples have been established, such as *t*-test, limma (Gentleman et al., 2004), edger (Robinson et al., 2010), and DEseq2 (Love et al., 2014). However, these methods tend to consider only the linear relationship (differential expression) between gene expression and phenotype, but may overlook nonlinear relationships (as shown in **Figure 1**) because gene expression may differ between population groups or indeed between individuals, and can also vary as a patient’s status changes. As shown in **Figure 1**, both high and low levels of *IGLC1* gene expression are found in non-tumor samples. The *IGLC1* gene would thus generally be overlooked in cancer studies, with it giving a large *p*-value in the *t*-test (0.48). However, the expression of this gene is actually very useful to discriminate between control subjects and patients affected by prostate cancer.

The maximal information coefficient (MIC) was proposed to capture a wide range of associations of two variables, in both linear and nonlinear relationships (Reshef et al., 2011). Owing to its generality, MIC is becoming widely accepted in scientific research (Zhang et al., 2014), and is also used to analyze large biological datasets (Rau et al., 2013; Wang and Zhao, 2015; Wang et al., 2016). However, even with MIC, it is difficult to identify genes with a nonlinear expression pattern as the genes giving the most strongly supported hits are linearly expressed. This makes MIC less practical for the exploration of nonlinear informative genes in next-generation sequencing datasets such as those obtained by RNA-seq.

We developed a novel nonlinearity measure, normalized differential correlation (NDC), which could efficiently find nonlinearly expressed genes in RNA-seq datasets. We verified its validity on six real-world, publicly available cancer RNA-seq datasets (for details on the datasets, see **Table 1**). The results showed that it could discover most genes that were nonlinearly expressed across groups of samples, even though these genes could not be identified by popular differential expression analysis approaches. We also validated these genes using classification performance, gene function analysis, and survival analysis. As expected, the results confirmed that the expression of these genes is related to the phenotype.

**Table 1:** Six binary-class RNA-seq datasets.

Cancer types	No. of patients	No. of gene
Breast Cancer (BRCA)	114	20,530
Kidney Clear Cell Carcinoma (KIRC)	72	20,530
Liver Cancer (LIHC)	50	20,530
Lung Adenocarcinoma (LUAD)	58	20,530
Lung Cancer (LUNG)	109	20,530
Lung Squamous Cell Carcinoma (LUSC)	51	20,530

## Datasets and Methods

### Datasets

The details of the six gene expression RNA-seq datasets are summarized in **Table 1**. Level 3 data of TCGA were downloaded from the UCSC Xena platform (Goldman et al., 2019). For each dataset, the patient samples do not contain paracarcinoma tissue were excluded.

### Methods

MIC can capture dependence between pairs of variables, including both functional and nonfunctional relationships. However, the ApproxMaxMI method provided by Reshef et al. (2011) results in a larger MIC score for paired variables under finite-sample conditions (Chen et al., 2016). Here, we use the improved algorithm ChiMIC to calculate the MIC value (Chen et al., 2016). The NDC score for a pair of data series *x* (gene) and *y* (phenotype) is defined as follows:

(1) $$NDC(x,y)=\frac{MIC(x,y)-R^{2}(x,y)-Thre_{MIC}}{\left|R(x,y)\right|}$$

Here, *R* ^2^ (*x*,*y*) and |*R*(*x*,*y*)| are the determination coefficient and absolute value of the correlation coefficient, respectively. For a discrete phenotype, *R* ^2^(*x*,*y*) and *R*(*x*,y) are effective only for a binary class. *Thre_MIC_*, the confidence limit at level *p* = 0.05 based on a given sample size, can be calculated as follows:

Step 1: Expression data of one gene (*x_i_*) are selected at random from the expression matrix and the sample order is shuffled;

Step 2: The MIC value between initial categorization labels (normal or diseased) and shuffled expression data is calculated;

Step 3: This process is repeated 1,000 times or more and these MIC values are sorted in ascending order. The MIC value at the 95% fractile is denoted as *Thre_MIC_*.

The NDC score is the normalized difference between nonlinear measure (MIC) and linear measure (*R* ^2^) by |R|. Because the MIC value is suspected of being large under finite-sample conditions, we use *MIC*(*x*,*y*)-*Thre_MIC_* to rule this out. We use the nonlinear score (“*MIC*(*x*,*y*)-*Thre_MIC_*“) minus the linear score (*R* ^2^) to exclude the linearly expressed genes. Lastly, we use *R*(*x*,*y*) as the background normalized to exclude the genes that are irrelevant to the categorization labels. Therefore, a higher NDC score indicates a strong nonlinear association, but a weak linear correlation.

**Figure 2:**
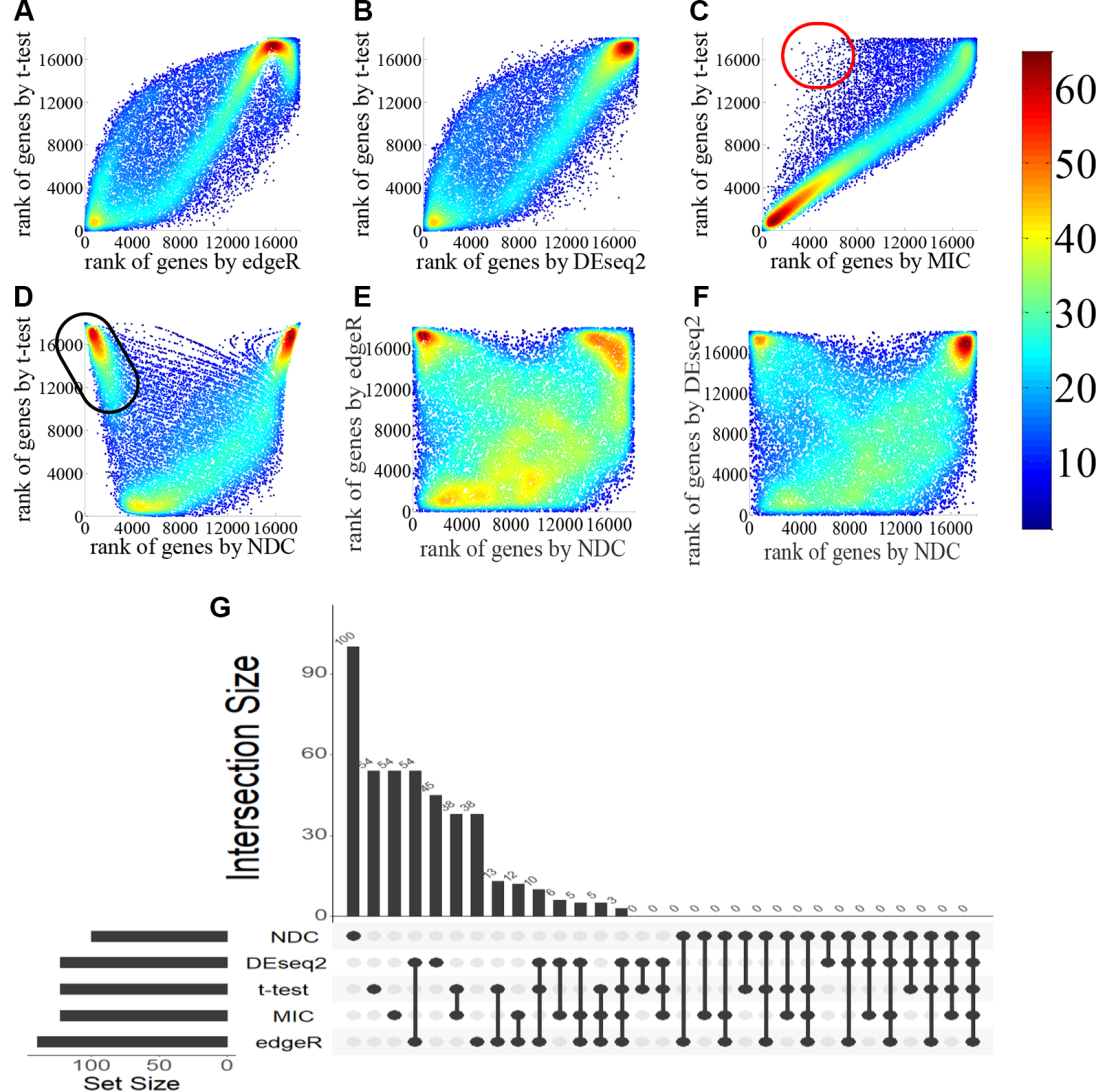
Comparison of gene ranking by *t*-test, edgeR, DEseq2, maximal information coefficient (MIC), and normalized differential correlation (NDC) for the lung squamous cell carcinoma (LUSC) dataset. In **(A–F)**: the color indicating data density; The red and black circles are just approximate region to visualize that, there are some genes can be highlighted by DNC methods, but cannot be highlighted by t-test. **(G)** overlaps of the top 100 genes selected by five methods.

**Figure 3:**
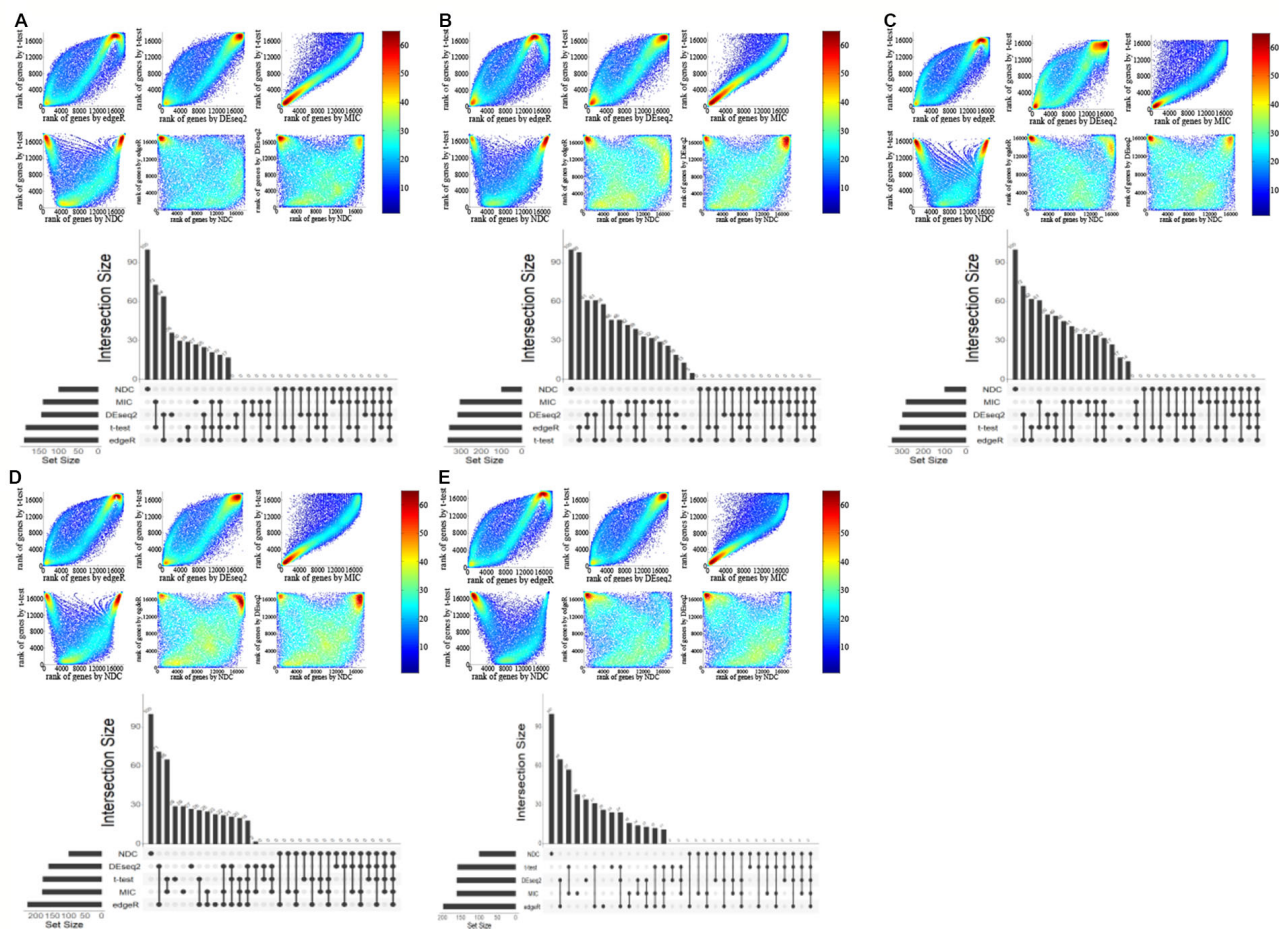
Comparison of genes selected by *t*-test, edgeR, DEseq2, maximal information coefficient (MIC), and normalized differential correlation (NDC) on five other datasets. **(A)** BRCA, **(B)** KIRC, **(C)** LIHC, **(D)** LUAD, **(E)** LUNG.

In **Figures 2A–D**, the genes are ranked by the NDC score, as well as the corresponding result in **Figure 3**. In other results, we first rank the genes in descending order of NDC score; then, we further sort the 1,000 most highly ranked genes in descending order by MIC value to obtain the most important nonlinear genes (top genes).

## Results and Discussion

### Identification of Nonlinearly Expressed Informative Genes

Four popular differential expression analysis methods, namely, *t*-test, edgeR, DEseq2, and MIC, were selected here for comparison with the NDC measure. We took the typical linear method, *t*-test, as a benchmark. **Figures 2A–F** illustrate and compare the rank of each gene between the *t*-test and the other methods for lung squamous cell carcinoma (LUSC). As shown in **Figure 2C**, most of the top genes highlighted by MIC are linearly expressed. There are also some genes (red circled area) that can be detected by MIC but not by *t*-test, edgeR, and DEseq2; these can be defined as nonlinearly expressed genes. However, these nonlinearly expressed genes cannot be identified as important by MIC as it confers a low ranking on them. As indicated in **Figures 2D–F**, only the NDC measure can efficiently identify the importance of these nonlinearly expressed genes (such as the black circled area). We also used the rank correlation coefficient to quantify the results by different methods, as shown in **Table 2**. **Figure 2G** illustrates the overlaps of the top 100 genes selected by five methods. There are many overlaps among the top 100 genes selected by the *t*-test, edgeR, DEseq2, and MIC methods, but no overlap between the NDC measure and the other methods. The same results were also obtained for five other datasets (**Figure 3**). We therefore deduce that the NDC measure is able to identify important nonlinearly expressed genes, while the other four reference methods could only discover linearly expressed ones.

**Table 2:** Rank correlation coefficient among five methods.

Methods	t-test	EdgeR	DESeq2	MIC
EdgeR	0.763			
DESeq2	0.778	0.967		
MIC	0.870	0.654	0.668	
NDC	**0.080**	**0.033**	**0.045**	0.494

**Figure 4:**
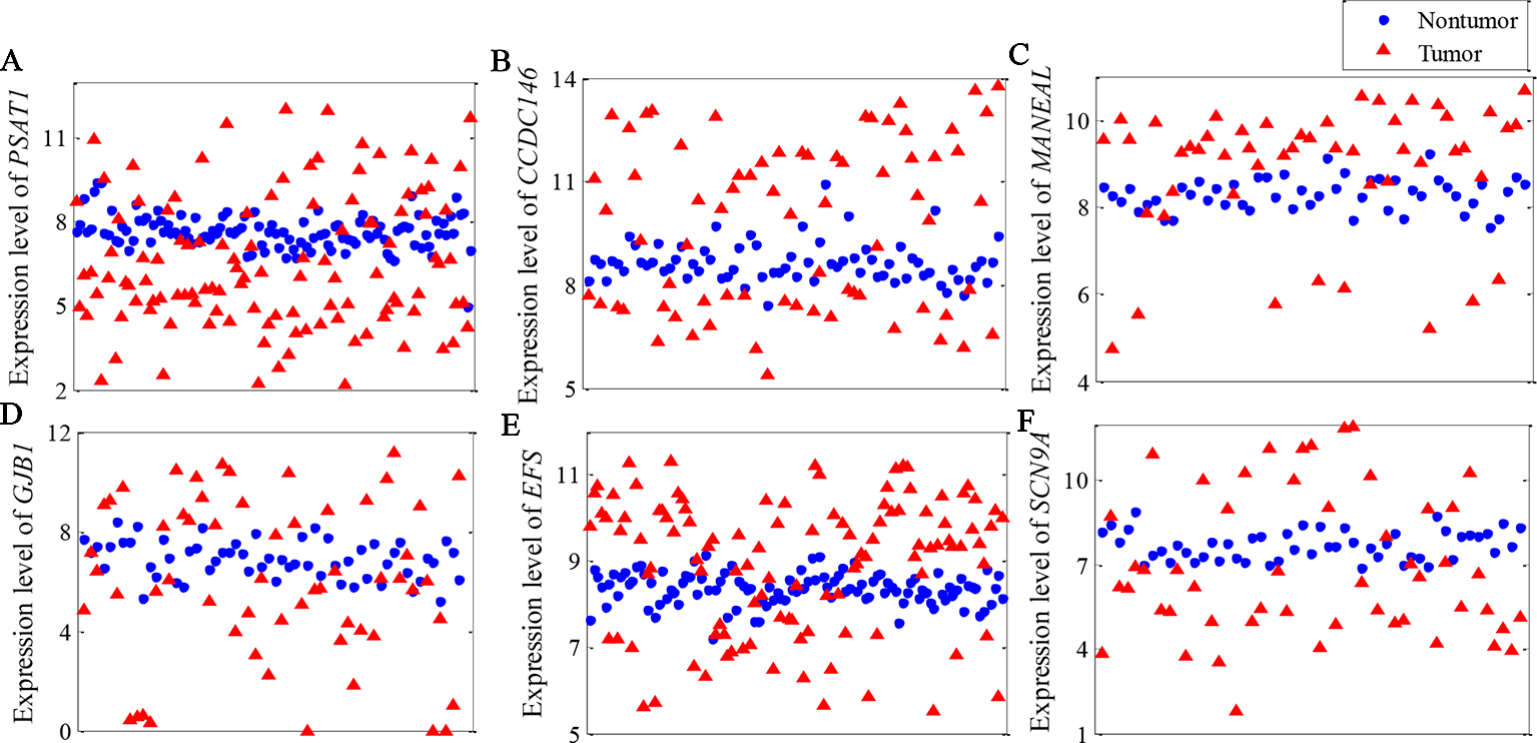
Expression pattern of top gene highlighted by the normalized differential correlation (NDC) measure for each dataset. **(A)** BRCA, **(B)** KIRC, **(C)** LIHC, **(D)** LUAD, **(E)** LUNG, **(F)** LUSC.

**Figure 4** illustrates the top gene highlighted by the NDC measure for each dataset. The expression patterns of these genes are clearly the same as in **Figure 1**. From a data-driven perspective, these genes have high power to discriminate between the two classes. However, they achieve a low ranking with the reference methods. For example, with regard to PSAT1 for BRCA (**Figure 4A**), the rankings by *t*-test, DESeq2, edgeR, and MIC are 8,583rd, 4,642nd, 4,292nd, and 947th, respectively. The details of the rankings by the reference methods for the top genes highlighted by NDC are shown in **Table 3**.

**Table 3:** The ranking order by reference methods for the top gene of six datasets highlighted by normalized differential correlation (NDC).

Gene symbol	t-test	edgeR	DEseq2	MIC
PSAT1	8,583	4,642	4,292	947
CCDC146	10,504	4,738	5,525	1,081
MANEAL	8,983	6,521	7,713	216
GJB1	13,072	6,049	5,969	1,336
EFS	11,207	7,742	8,559	957
SCN9A	13,660	9,275	7,842	1,091

**Figure 5:**
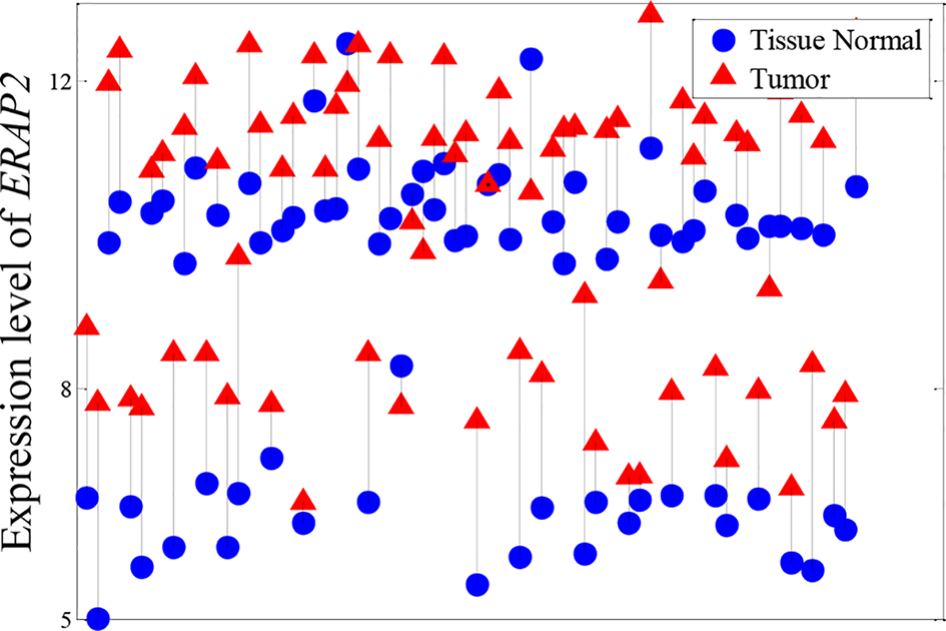
Expression pattern of ERAP2 in KIRC. Tumor samples and paracarcinoma tissue samples from the same patient are connected by a black line.

Besides the expression patterns shown in **Figure 4**, another pattern was identified in KIRC, as shown in **Figure 5**. We found that the expression level of ERAP2 was stratified into four levels. Essentially, ERAP2 is a differentially expressed gene here, with 27 out of 72 patients having lower ERAP2 expression levels. ERAP2 is a proteolytic enzyme that acts in the endoplasmic reticulum, where it plays a central role in the trimming of peptides for presentation by MHC class I molecules (MHC I) (Serwold et al., 2002; Saveanu et al., 2005). Forloni et al. (2010) reported that MHC I and ERAP2 are under the control of NF-κB through enhancer A in human neuroblastoma cells. Gadalla et al. (2013) also found that ERAP2 interacts with epithelial cell adhesion molecule (EpCAM) in breast cancer cells; EpCAM is a well-known epithelial and cancer cell “marker” (Tandon et al., 1990; Spizzo et al., 2006; Baeuerle and Gires, 2007). These patients in this study were also divided into two groups regarding their expression levels of ERAP2; this indicated that the expression of ERAP2 was strongly associated with the tumor grade (p-value of chi-squared test of 0.0084). Those in the group with high expression of ERAP2 have poorly differentiated KIRC (**Table 4**). All of this evidence suggests that ERAP2 may play a regulatory role in cancer. However, only the NDC method was able to identify the importance of ERAP2 (ranking: 14th); in contrast, all of the reference methods failed to identify the importance of this gene, with the rankings by *t*-test, DESeq2, edger, and MIC being 10,567th, 4,819th, 4,382, and 2,561th, respectively.

**Table 4:** Contingency table for expression of ERAP2 and tumor grade in KIRC.

	Low expression	High expression
G1 or G2	16	10
G3 or G4	10	29

G1: well differentiated, G2: moderately differentiated, G3: poorly differentiated, G4: undifferentiated. Seven patients (TCGA-B8-5552, TCGA-CJ-5679, TCGA-CJ-5680, TCGA-CJ-5681, TCGA-CW-5591, TCGA-CZ-5456, TCGA-CZ-5469) are excluded due to inconsistent expression patterns.

### Classification Accuracy

To measure the impact of the nonlinearly expressed genes selected by NDC on the classification, we examined the classification accuracy on the six datasets with a supported vector classifier (SVC). SVC was performed using LIBSVM (Chang and Lin, 2011), which is available at http://www.csie.ntu.edu.tw/~cjlin/libsvm/index.html. Rigorous 10-fold cross-validation was used to evaluate the performance of the various gene selection algorithms. The dataset was randomly divided into 10 subsets. For each run, 9 out of 10 subsets were selected to be the training set and the remaining samples were used as test samples. For a fair comparison between gene selection algorithms, the training and test sets for each run were kept the same for all algorithms. In this section, in contrast to the case in the other sections, we could obtain 10 gene subsets for each method on each dataset.

**Figure 6:**
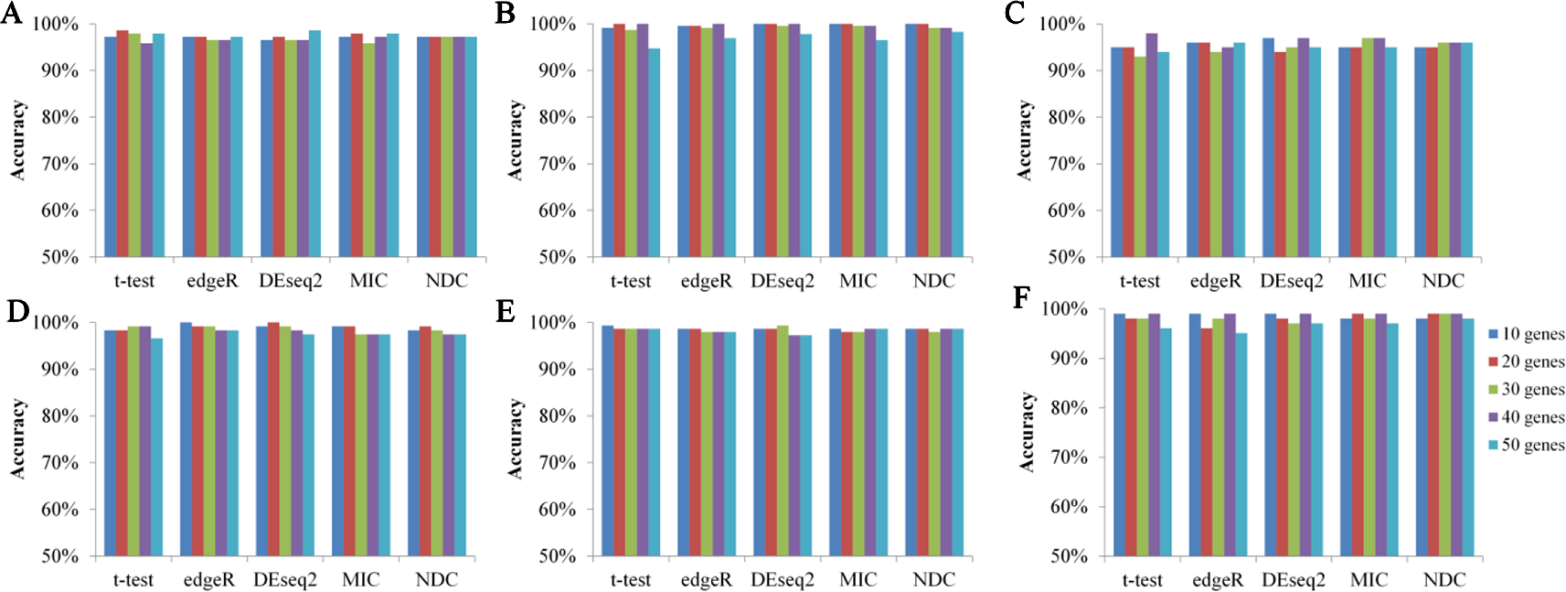
Prediction accuracy of five methods on six datasets based on supported vector classifier (SVC). **(A)** BRCA, **(B)** KIRC, **(C)** LIHC, **(D)** LUAD, **(E)** LUNG, **(F)** LUSC.

**Figure 6** illustrates the 10-fold cross-validation prediction accuracy using the top 10, 20, 30, 40, and 50 genes selected by NDC, as well as by *t*-test, edgeR, DEseq2, and MIC. The results reveal comparable accuracy for the five methods. This indicates that analysis of these nonlinearly expressed genes has sufficient ability to distinguish samples from cancerous tissue and from paracarcinoma tissue, *via* a machine learning approach.

### Biological Interpretation of the Nonlinearly Expressed Genes

#### Enrichment Analysis of Pathways and Biological Processes

**Figure 7:**
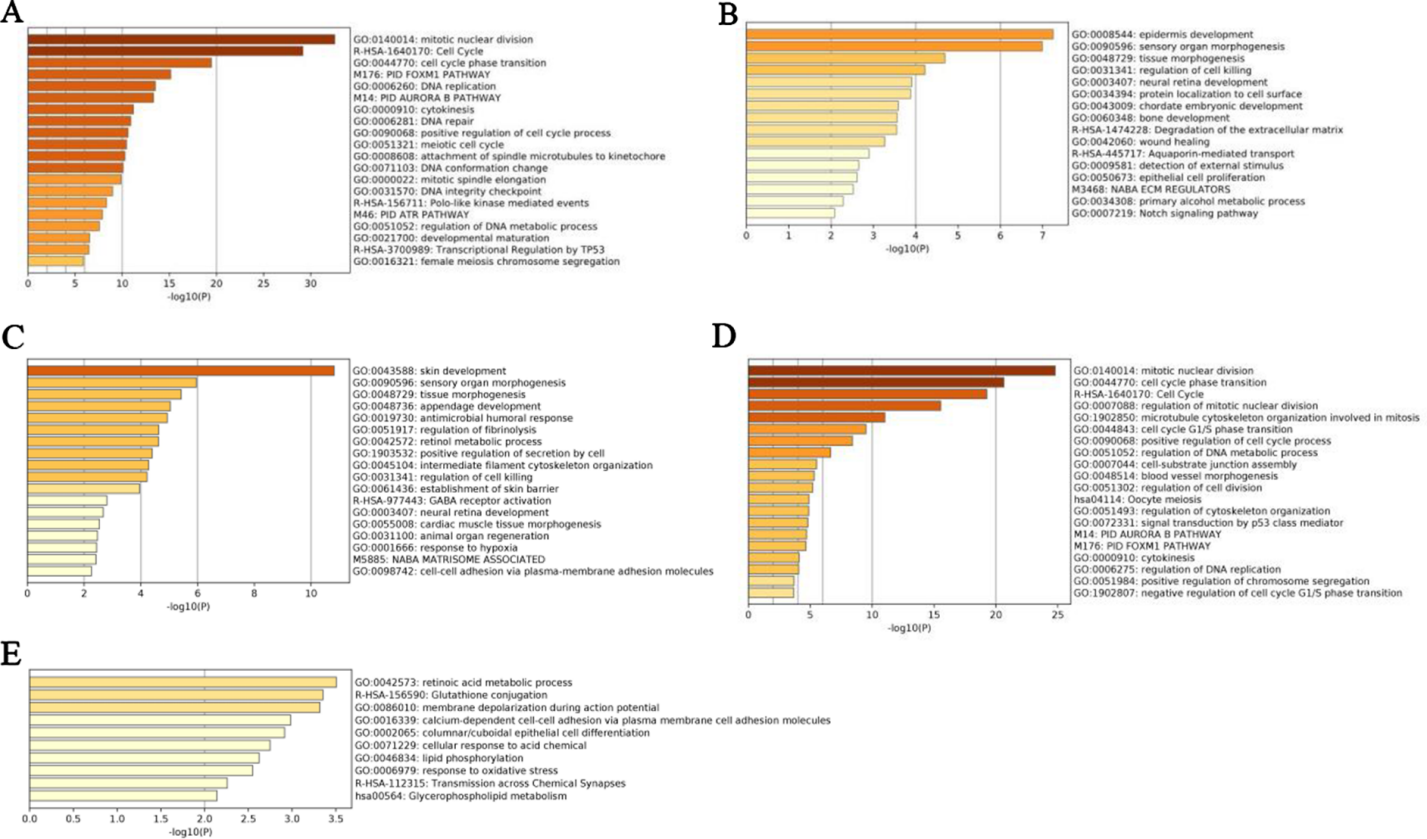
Enrichment analyses of pathways and biological processes by top 100 genes selected by five methods for lung squamous cell carcinoma (LUSC) dataset. **(A)** *t*-test, **(B)** edgeR, **(C)** DEseq2, **(D)** maximal information coefficient (MIC), **(E)** normalized differential correlation (NDC).

We further validated these top 100 genes selected by five methods using the LUSC dataset as an example, according to Metascape (A Gene Annotation & Analysis Resource) (Zhou et al., 2019), as shown in **Figure 7**. We found that these nonlinearly expressed genes selected by NDC (**Figure 7E**) were enriched in the ten terms, and these terms have no overlap between other terms enriched by reference methods. However, for the glutathione conjugation term, Fletcher et al. (2015) reported that glutathione-S-transferases (GSTs) catalyze the conjugation of glutathione with toxic oxidant compounds and were associated with acute and chronic inflammatory lung diseases. For the glycerophospholipid metabolism term, phosphoenolpyruvate carboxykinase (PEPCK) has been shown to promote cancer cell survival under conditions of nutrient deprivation, a typical feature in solid cancers, as well as cancer growth (Méndez-Lucas et al., 2014; Leithner et al., 2015; Montal et al., 2015; Vincent et al., 2015). Recently, Leithner et al. (2018) further showed that mitochondrial PEPCK (PEPCK-M) mediates the synthesis of glycerol phosphate from noncarbohydrate precursors, and that PEPCKM is needed to maintain the levels of glycerophospholipids, major constituents of biomembranes, in starved lung cancer cells.

#### Gene Module Analysis

**Figure 8:**
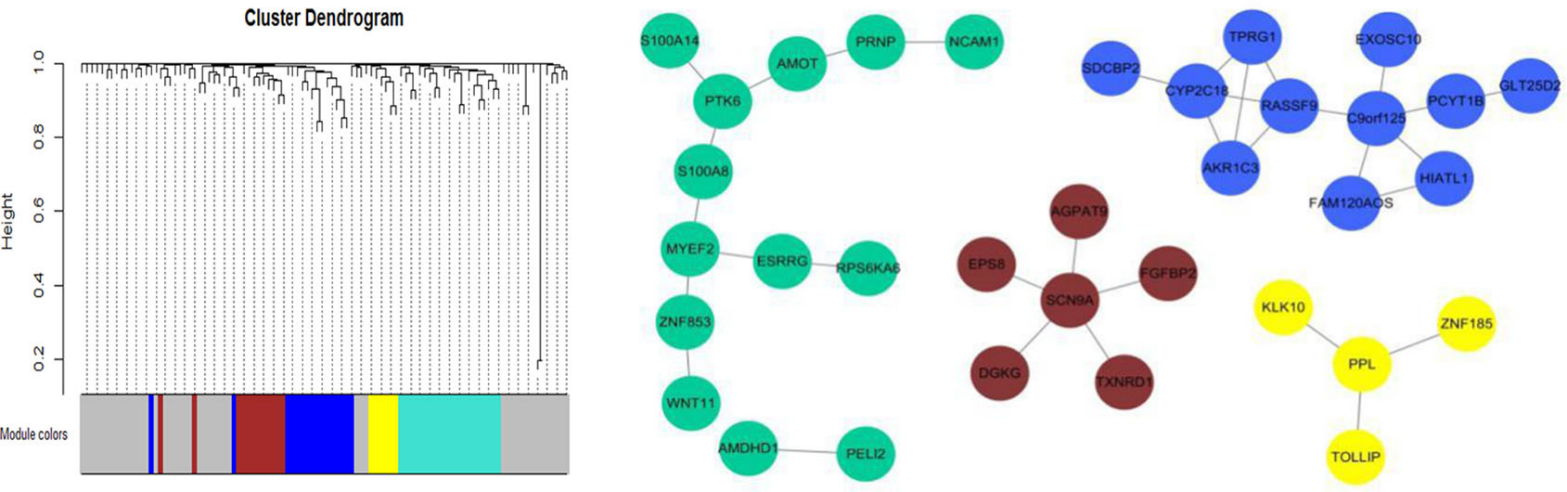
Weighted gene coexpression network analysis (WGCNA) for the top 100 nonlinearly expressed genes in the lung squamous cell carcinoma (LUSC) dataset. Power is set to 5 and the threshold of TOM is set to 0.05.

We further identified four co-expressed gene modules (as shown in **Figure 8**) by weighted gene coexpression network analysis (WGCNA) (Langfelder and Horvath, 2008) for the top 100 nonlinearly expressed genes in the LUSC dataset. For the hub gene SCN9A (Aliases: Nav1.7), which was also selected as the top gene by the NDC method. Campbell et al. (2013) found that the inhibition of Na_v_1.7 activity or expression could reduce H460 non-small cell lung carcinoma (NSCLC) cell invasion by up to 50%; this indicated that functional expression of the subunit Na_v_1.7 promotes the invasion of H460 NSCLC cells. For the hub gene PPL, the corresponding protein was reported to interact with AKT1 protein (van den Heuvel et al., 2002; Wang et al., 2008), suggesting its possible role as a localization signal in AKT1-mediated signaling. Notably, many studies have shown that aberrant Akt activation is associated with the development of many tumors (Steelman et al., 2008; Jin et al., 2019). Both David et al. (2004) and Lim et al. (2007) reported that p-Akt overexpression can be used as an indicator of poor prognosis in NSCLC.

#### Survival Analysis

**Figure 9:**
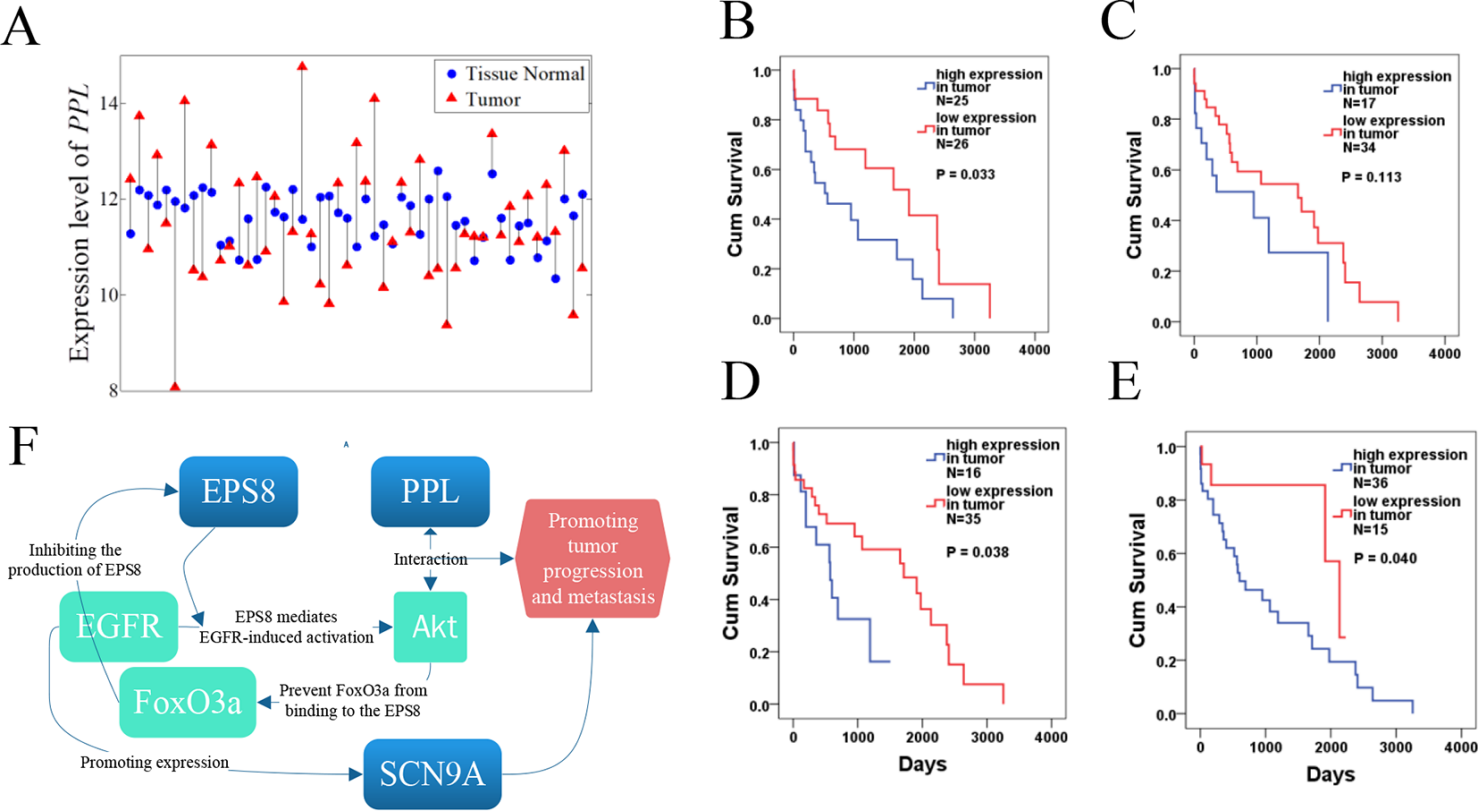
Survival analysis of PPL, SCN9A, EPS8, and MTAP for the lung squamous cell carcinoma (LUSC) dataset. **(A)** Expression pattern of PPL in LUSC. Tumor samples and paracarcinoma tissue samples from the same patient are connected by a black line. **(B–E)** Results of survival analysis for PPL, SCN9A, EPS8, and MTAP. **(F)** Functional network for PPL, SCN9A, and EPS8.

For the nonlinearly expressed genes, as shown in **Figure 9A**, the patients were divided into two groups depending on their expression pattern. In the case of the PPL gene in the LUSC dataset, it showed higher expression in tumor tissue for 26 patients and in paracarcinoma tissue for 25 patients. We further compared the overall survival of the two groups of patients, with the results indicating that some nonlinearly expressed genes were strongly associated with overall survival, such as PPL (hub gene for WGCNA), SCN9A (hub gene for WGCNA and the first ranked gene for NDC), EPS8 (second ranked gene for NDC), and MTAP (fourth ranked gene for NDC) for the LUSC dataset. As shown in **Figures 9B–E**, patients with the high expression of PPL in tumor tissue compared with the level in paracarcinoma tissue had significantly shorter overall survival (p = 0.033). SCN9A and EPS8 were the top 2 ranked genes selected by the NDC method, exhibiting similar patterns of expression (as shown in **Figure 8**). The expression of SCN9A was not significantly associated with overall survival (p = 0.113), but that of EPS8 was p = 0.038. It has been reported that EPS8 is strongly associated with tumor progression and metastasis (Wang et al., 2009; Mitra et al., 2011; Jeganathan et al., 2016; Fang et al., 2017). Furthermore, we found that PPL, SCN9A, and EPS8 are functionally related, as shown in **Figure 9F**. EPS8 is a crucial molecule that mediates EGFR-induced activation of Akt (Innocenti, 2003) and PPL proteins as a localization signal in AKT1-mediated signaling by interacting with AKT1 (van den Heuvel et al., 2002; Wang et al., 2008). Akt activation is well known to be associated with the development of many tumors (Steelman et al., 2008; Jin et al., 2019), and it can also prevent FoxO3a, a tumor suppressor, from binding to EPS8. Moreover, it was reported that EGFR, *via* a U0126-sensitive ERK1/2 pathway, controls the transcriptional upregulation of SCN9A to promote cellular invasion in NSCLC cell lines (Campbell et al., 2013). MTAP, a tumor suppressor gene, has been reported to be associated with the overall survival of NSCLC patients, which is consistent with our results (p = 0.04) (Su et al., 2014). Furthermore, the expression pattern of MTAP is strongly correlated with the vital status of LUSC patients (p = 0.002). A total of 28 out of 36 patients with the high expression of MTAP in tumor tissue compared with the level in paracarcinoma tissue died, but only 4 out of 15 with a low MTAP expression level in tumor tissue did so.

## Conclusion

In this paper, we propose a novel nonlinearity measure named NDC to efficiently identify nonlinearly expressed genes in transcriptome datasets. Validation using six real-world cancer datasets revealed that the NDC method could identify nonlinearly expressed genes that were overlooked by *t*-test, MIC, edgeR, and DEseq2, although MIC could capture nonlinear correlations. The results regarding the classification accuracy indicate that these genes have sufficient ability to distinguish cancer and paracarcinoma tissue samples. Moreover, the results of biological interpretation of these genes also suggest that some of them are involved in key functional pathways associated with cancer progression and metastasis. All of this evidence suggests that these nonlinearly expressed genes may play central roles in regulating cancer progression. Interestingly, as shown in **Figures 4** and **5**, analysis of these nonlinearly expressed genes proved that genes could be expressed in different patterns in different patients. This explains the need for the development of precision medicine, but also the challenges associated with this. Not all of the top-ranked nonlinearly expressed genes or hub genes found here have previously been reported to correlate with cancer progression and metastasis, but the NDC method suggests their importance as informative genes. The approach presented here suggests that these genes warrant attention as potential targets for therapy and disease risk predictors, as well as for their ability to achieve a clinical diagnosis and evaluate therapeutic efficacy.

## Data Availability Statement

Publicly available datasets were analyzed in this study. This data can be found here: https://xenabrowser.net/datapages/ and the NDC algorithm can be found here: https://github.com/chenyuan0510/normalized-differential-correlation-NDC-.git.

## Author Contributions

All authors contributed to the methodology proposal, analysis of results, writing, and review of the manuscript.

## Funding

This work was supported by the National Natural Science Foundation of China (61701177); Hunan Provincial Natural Science Foundation (2018JJ3225); Science foundation open project of Hunan Provincial Key Laboratory of Crop Germplasm Innovation and Utilization (18KFXM08); and Hunan Provincial Research learning and innovative experimental project for college students (SCX1826).

## Conflict of Interest

The authors declare that the research was conducted in the absence of any commercial or financial relationships that could be construed as a potential conflict of interest.
